# Operative Efficiency Among Robotic-, Navigation-, and Fluoroscopy-Assisted Systems in 1- and 2-Level Open Transforaminal Lumbar Interbody Fusion

**DOI:** 10.7759/cureus.108481

**Published:** 2026-05-08

**Authors:** Evan Brown, Micah Smith

**Affiliations:** 1 Spine Surgery, Indiana University School of Medicine, Indianapolis, USA; 2 Spine Surgery, SpineONE, Orthopaedics Northeast, Fort Wayne, USA

**Keywords:** fluoroscopy-assisted surgery, learning curve, lumbar spine surgery, navigation-assisted surgery, operative time, pedicle screw placement, robotic-assisted surgery, surgical guidance systems, technology adoption, transforaminal lumbar interbody fusion (tlif)

## Abstract

Background: Navigation-assisted (NA) and robotic-assisted (RA) systems are being employed as guidance systems over traditional fluoroscopy-assisted (FA) systems to enhance surgical accuracy and safety. These systems allow surgeons to plan screw trajectories based on patient-specific anatomy and obtain real-time tracking of navigated instruments. This study sought to analyze procedural efficiency and outcomes with these systems.

Methods: A retrospective chart review was conducted for patients who underwent 1- or 2-level open transforaminal lumbar interbody fusion (TLIF) as a primary procedure by a single surgeon from 2014 to 2024. Patients needed to be ≥18 years, diagnosed with lumbar degenerative or deformity conditions. Operative time (OT) (skin to closure) in minutes, length of stay (LOS) in days, estimated blood loss (EBL) in mL, and fluoroscopy time (FT) in seconds were analyzed.

Results: The study included 170 patients operated at 1-level (118, 20, and 32 cases for FA, NA, and RA systems, respectively) and 74 patients operated at 2-levels (44, nine, and 21 cases for FA, NA, and RA systems, respectively). There was no significant difference in operative efficiency (OT, LOS, EBL, FT) between NA and RA in both 1- and 2-level procedures. The RA group showed significantly less OT, EBL, and LOS than the FA group for both 1- and 2-level procedures. When looking at a narrow transitional period of robotic adoption, OT and LOS were not markedly different between the first 9-10 RA cases and the last 9-10 NA cases.

Conclusion: In both 1- and 2-level open TLIF procedures, the NA and RA systems showed similar outcomes to each other and statistically significant improvements in OT and EBL compared to the FA system. An experienced NA system user transitioning to a RA system did not experience reductions in operative efficiency.

## Introduction

Lumbar spine pathology is a prevalent condition affecting a considerable portion of the global population [1,2]. Common etiologies include disc degeneration, stenosis, and spondylolisthesis, which lead to the compression of the thecal sac and the nerve roots. A common method to treat patients with symptomatic lumbar degenerative conditions who have failed to improve through nonoperative treatments is lumbar interbody fusion (LIF) surgery [3]. One dominant method for LIF is transforaminal lumbar interbody fusion (TLIF), which has been performed for decades and has been shown to be effective in improving back and radicular pain and enhancing patients’ quality of life [4-6].

Placement of pedicle screws during a TLIF procedure can be performed using various techniques to improve surgical outcomes. Currently, there are three main techniques: fluoroscopy-assisted (FA) (the oldest), navigation-assisted (NA) (intermediate), and robotic-assisted (RA) (the most recent) [7-9]. The adoption of NA and RA systems has grown quickly. They have yielded highly accurate screw placement, achieving success rates of 84-96%, compared to the 79-94% typically seen in FA systems [10-12]. Furthermore, they have demonstrated a reduction in complication rates from approximately 14.97% in freehand surgery to as low as 4.83% [10,11]. Additionally, these new techniques have been shown to reduce radiation exposure by 70-90% per case and mental workload (lower National Aeronautics and Space Administration Task Load Index (NASA-TLX) scores) for the surgical team, yielding comparable operation time, 75-140 mL less blood loss per case, and up to 1.5 shorter hospital stay for patients than traditional FA systems [13-15].

Current literature shows that proficiency with the RA technique can be achieved after completing 25 cases with subsequent improvements in the time required for robot setup, registration, fluoroscopy, and screw insertion [16]. Such initial inefficiencies may be a barrier to more widespread adoption of enabling technologies in spinal surgery. However, there are limited articles in the literature evaluating the impact of incorporating these newer systems on operative efficiency compared to the well-established FA technique [17]. This gap in the literature is particularly notable regarding the initial adoption phase, where the lack of granular data on early-stage usability hinders the field’s understanding of the immediate barriers surgeons face when transitioning between guidance modalities.

This study aimed to compare the operative efficiency of the three different guidance systems when adopted sequentially by a single surgeon for performing 1- and 2- level open TLIF surgeries. Operative efficiency comprises four components: operative time (OT), fluoroscopy time (FT), blood loss, and length of stay (LOS). These metrics were selected for their widespread use and critical relationships to hospital economics and patient safety. Furthermore, this study aimed to evaluate how the initial operative efficiency is affected when an experienced NA system user switches to an RA system.

## Materials and methods

Study design and data collection

The study was conducted through a retrospective chart review performed by a single surgeon at Parkview Orthopedic Hospital, a specialty orthopedic surgery hospital in Fort Wayne, USA, that provides both inpatient and outpatient services. It included all patients who met the following inclusion criteria: (i) ≥18 years of age at the date of surgery, (ii) underwent 1- or 2-level open TLIF surgery between 2014 and 2024, (iii) diagnosed with any of the following conditions: symptomatic anterolisthesis, retrolisthesis, deformity, spinal stenosis, radiculopathy, herniated nucleus pulposus, or recurrent stenosis, (iv) had a minimum of four weeks of postoperative follow-up data, providing a sufficient window to evaluate immediate operative effects and early clinical recovery rather than long-term procedure related outcomes. This study was conducted after qualifying for the institutional review board (IRB) exemption from Parkview Health IRB. To have the most homogenous cohorts, cases involving revision of prior spinal instrumentation were excluded due to the additional surgical time for removal, which tends to be variable depending on the type and complexity of the instrumentation removed.

Data collection included patient demographics (i.e., age, gender, race, body mass index (BMI), and comorbidities), surgical efficiency details (i.e., type of assisted system used, LOS, OT, estimated blood loss (EBL), and FT), and 30-day revision surgery. OT was defined as the time from the start of skin incision to the start of skin closure. EBL was based on the volume of blood captured in the cell saver and the blood extracted from sponges, which were rinsed to recover the absorbed blood. FT was defined as the total duration of FT during the operation recorded by the C-arm (OEC 9900 Elite C-arm; GE Healthcare, Chicago, USA).

In addition to the analysis of the full cohorts described by the inclusion and exclusion criteria, a secondary sub-analysis was conducted to determine whether there were differences between NA and RA systems in a narrower transitional period immediately following RA system adoption. For 1-level cases, the transitional period was defined by the first 10 cases in the RA group, which were compared to the last 10 cases in the NA group. For 2-level cases, the transitional period was limited to nine cases per group, as limited by the total number of cases in the NA group.

Surgical technique

The open TLIF surgery was performed by placing the patient in the prone position, and the surgical level was confirmed. A posterior midline incision was made to expose the affected vertebral level. Subperiosteal dissection reveals the lamina and facet joints, allowing a unilateral facetectomy on the symptomatic side for access to the disc space. Any necessary decompression was performed to relieve nerve compression. The intervertebral disc was then accessed, and the disc material was removed. Endplates were carefully prepared, and an interbody cage packed with autograft (collected from the facetectomy) was inserted into the disc space to restore disc height and maintain alignment. The open incision provided clear visualization of the anatomy throughout the procedure, enabling thorough direct decompression, safe avoidance of nerve roots and the thecal sac, complete discectomy and endplate preparation, and precise selection and placement of an interbody spacer for fusion.

Pedicle screws were placed bilaterally using either the FA, NA, or RA systems. The FA free-hand technique was accomplished using a starting spot at the junction of the lateral border of the pars interarticularis and the midpoint of the transverse process. A ball-tipped probe was passed through the pedicle to ensure no cortical breaches, and then the screw was placed. The NA system (SpineMap Go software along with NAV3i Platform; Stryker Corp., Kalamazoo, USA) was used to navigate fixation system instruments, and the C-arm was used to take intraoperative fluoroscopic images to visualize the patient's anatomy and monitor the navigated instruments during surgery. The RA system (ExcelsiusGPS; Globus Medical, Inc., Audubon, USA) was used to provide real-time spatial guidance for visualization of the screw trajectory while placing the pedicle screws through the robotic arm. When using the RA system, the robotic arm was aligned to the surgeon’s pre-planned pedicle screw trajectory, enabling placement of the screws according to the planned trajectory. With all techniques, once the screws were placed, rods were secured across the screws to stabilize the segment. Final anteroposterior (AP) and lateral X-ray images were taken to confirm proper implant placement and alignment. Finally, the incision was closed with staples or sutures and covered with a sterile dressing.

Statistical analysis

Continuous variables were summarized using mean and standard error, while nominal data were reported as frequencies and percentages. To evaluate differences in continuous variables across the three study groups, the Kruskal-Wallis test was employed. Where significant differences were identified, post-hoc pairwise comparisons were performed to isolate specific group differences. Categorical variables were analyzed using Fisher’s exact test. All tests were two-tailed with a significance threshold of p<0.05 and a 95% confidence level.

## Results

The surgeon (senior author) treated roughly 1500 patients with FA (2014-2020), 172 patients with NA (2021), and 378 patients with RA (2022-2024) between 2014 and 2024. From this operating history, 244 patients (170 patients treated with TLIF at 1-level and 74 patients treated at 2-level) met the eligibility criteria and were included in this study. The 1-level cases were segmented into the FA group (118 patients), the NA group (20 patients ), and the RA group (32 patients). Similarly, the 2-level cases were segmented into the FA group (44 patients), the NA group (nine patients), and the RA group (21 patients). Operative efficiency data were available for all cases with the exception of FT in 2.5% (3/118) and 2.3% (1/44) of FA patients and 15.6% (5/32) and 14.3% (3/21) of RA patients for 1- and 2-level cases, respectively.

Baseline characteristics

Patient data are summarized in Tables 1-2. For the 1-level cases, the mean age was 52.9, 54.3, and 50.4 years in the FA, NA, and RA groups, respectively. Male patients comprised 44.1%, 55.5%, and 59.4% in the FA, NA, and RA groups, respectively. Average BMI was 31.4 kg/m^2^ in the FA group, 30.7 kg/m^2^ in the NA group, and 33.2 kg/m^2^ in the RA group. For the 2-level cases, the mean age was 60.6 years in the FA group, 60.0 years in the NA group, and 58.8 years in the RA group. Gender data showed 56.8% male patients in the FA group, 66.7% in the NA group, and 71.4% in the RA group. Mean BMI was 31.8 kg/m^2^, 32.4 kg/m^2^, and 31.1 kg/m^2 ^in FA, NA, and RA groups, respectively.

**Table 1:** Demographics data for 1-level cases For getting the p-values, Kruskal-Wallis tests were run for age and BMI. Fisher’s exact tests were run for gender, race, and comorbidities. SE: standard error; BMI: body mass index

Parameter	Fluoroscopic (n=118)	Navigation (n=20)	Robotic (n=32)	p-values
Age (years) (mean ± SE)	52.9 ± 1.2	54.3 ± 2.6	50.4 ± 2.7	0.37
Gender (n, %)				
Male	52, 44.1%	11, 55.5%	19, 59.4%	0.25
Female	66, 55.9%	9, 45.0%	13, 40.6%	
BMI (kg/m^2^) (mean ± SE)	31.4 ± 0.6	30.7 ± 0.9	33.2 ± 1.1	0.27
Race (n, %)				
White	110, 93.2%	19, 95.0%	29, 90.6%	0.51
Black	3, 2.5%	0, 0.0%	3, 9.4%	
Asian	1, 0.9%	0, 0.0%	0, 0.0%	
Hispanic	2, 1.7%	1, 5.0%	0, 0.0%	
American Indian or Alaskan Native	2, 1.7%	0, 0.0%	0, 0.0%	
Comorbidities (n, %)				
Diabetes	22, 18.6%	3, 15.0%	5, 15.6%	0.54
Hypertension	61, 51.7%	13, 65.0%	13, 40.6%	
Cardiac issue	18, 15.3^	2, 10.0%	5, 15.6%	
Renal issue	5, 4.2%	1, 5.0%	1, 3.1%	
Respiratory issue	42, 35.6%	7, 35.0%	3, 9.4%	
Cancer	12, 10.2%	2, 10.0%	3, 9.4%	
Arthritis	27, 22.9%	9, 45.0%	12, 37.5%	
Osteoporosis	4, 3.4%	0, 0.0%	2, 6.3%	

**Table 2:** Demographics data for 2-level cases For getting the p-values, Kruskal-Wallis tests were run for age and BMI. Fisher’s exact tests were run for gender, race, and comorbidities. SE: standard error; BMI: body mass index

Parameter	Fluoroscopic (n=44)	Navigation (n=9)	Robotic (n=21)	p-values
Age (years) (mean ± SE)	60.6 ± 1.6	60.0 ± 3.8	58.8 ± 2.1	0.68
Gender (n, %)				
Male	25, 56.8%	6, 66.7%	15, 71.4%	0.15
Female	19, 43.2%	3, 33.3%	6, 28.6%	
BMI (kg/m^2^) (mean ± SE)	31.8 ± 0.9	32.4 ± 2.2	31.1 ± 1.2	0.87
Race (n, %)				
White	40, 90.9%	9, 100.0%	19, 90.4%	0.05
Black	4, 9.1%	0, 0.0%	1, 4.8%	
Asian	0, 0.0%	0, 0.0%	0, 0.0%	
Hispanic	0, 0.0%	0, 0.0%	1, 4.8%	
American Indian or Alaskan Native	0, 0.0%	0, 0.0%	0, 0.0%	
Comorbidities (n, %)				
Diabetes	11, 25.0%	4, 44.4%	6, 28.6%	0.77
Hypertension	32, 72.7%	6, 66.7%	12, 57.1%	
Cardiac issue	12, 27.3%	4, 44.4%	2, 9.5%	
Renal issue	2, 4.5%	2, 22.2%	2, 9.5%	
Respiratory issue	11, 25.0%	2, 22.2%	6, 28.6%	
Cancer	6, 13.6%	3, 33.3%	3, 14.3%	
Arthritis	16, 36.4%	3, 33.3%	12, 57.1%	
Osteoporosis	1, 2.3%	1, 11.1%	1, 4.8%	

Operative efficiency - NA vs. FA

**Figure 1:**
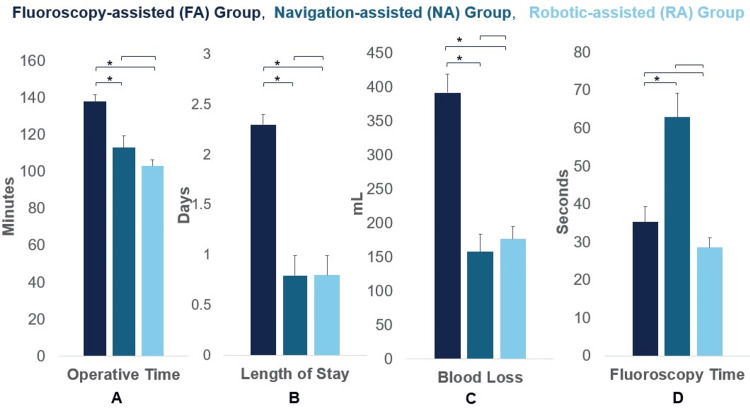
Operative efficiency results for 1-level cases Comparison of fluoroscopic, navigation, and robotic systems for single-level cases across four parameters: (A) operative time, (B) length of stay, (C) blood loss, and (D) fluoroscopy time. Data are presented as mean ± standard error. * indicates p<0.05, statistically significant.

**Figure 2:**
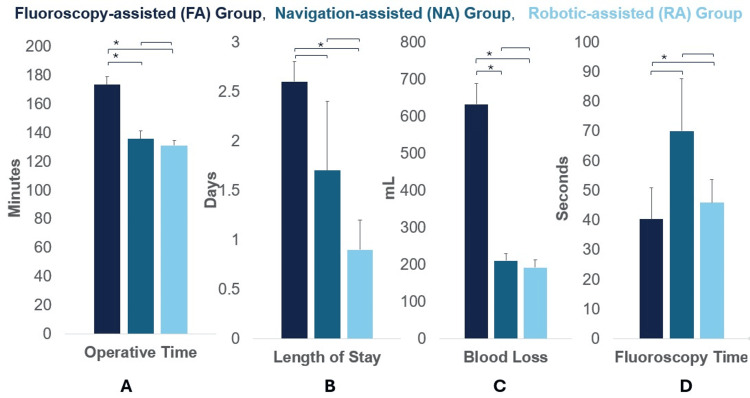
Operative efficiency results for 2-level cases Comparison of fluoroscopic, navigation, and robotic systems for two-level cases across: (A) operative time, (B) length of stay, (C) blood loss, and (D) fluoroscopy time. Data are presented as mean ± standard error. * indicates p<0.05, statistically significant.

In 1-level TLIF cases, the NA group experienced significantly shorter OT, reduced LOS, less EBL, and longer FT (113.0 ± 29.2 minutes, 0.8 ± 1.0 days, 158.0 ± 112.3 mL, 63 ± 68.9 seconds, respectively) than the FA group (137.7 ± 41.7 minutes, 2.3 ± 1.2 days, 391.9 ± 289.9 mL, 35.3 ± 42.6 seconds, respectively) (all p<0.05) (Figure 1). Similarly, in 2-level cases, the NA group experienced significantly shorter OT and less EBL (135.7 ± 16.4 minutes and 210.0 ± 55.2 mL, respectively) than the FA group (173.3 ± 36.6 minutes and 631.9 ± 374.5 mL, respectively) (all p<0.05). The FT tended to be longer (NA: 70.0 ± 53.0 seconds vs. FA: 40.3 ± 69.4 seconds) and LOS tended to be shorter (NA: 1.7 ± 2.3 days vs. FA: 2.6 ± 1.4 days) in the NA group in 2-level cases, but these differences were not significant (p=0.06 and p=0.09, respectively) (Figure 2).

Operative efficiency - RA vs. FA

In 1-level TLIF cases, the RA group experienced significantly shorter OT, reduced LOS, and less EBL (102.9 ± 18.9 minutes, 0.8 ± 1.1 days, and 176.7 ± 102.3 mL, respectively) than the FA group (137.7 ± 41.7 minutes, 2.3 ± 1.2 days, and 391.9 ± 289.9 mL, respectively) (all p<0.05). The FT tended to be shorter in the RA group (28.6 ± 13.5 seconds) than the FA group (35.3 ± 42.6 seconds), but this difference was not significant (p=0.23) (Figure 1). Similarly, in 2-level cases, the RA group experienced significantly shorter OT, reduced LOS, less EBL and longer FT (131.2 ± 16.3 minutes, 0.9 ± 1.3 days, 192.1 ± 94.5 mL, 45.8 ± 33.4 seconds, respectively) than the FA group (173.3 ± 36.6 minutes, 2.6 ± 1.4 days, 631.9 ± 374.5 mL, 40.3 ± 69.4 seconds, respectively) (all p<0.05) (Figure 2).

Operative efficiency - RA vs. NA

In 1-level TLIF cases, the OT and FT tended to be longer in the NA group (113.0 ± 29.2 minutes and 63 ± 68.9 seconds, respectively) compared to the RA group (102.9 ± 18.9 minutes and 28.6 ± 13.5 seconds, respectively), whereas LOS and EBL tended to be lower for NA group (0.8 ± 1.1 days and 158.0 ± 112.3 mL, respectively) versus the RA group (0.8 ± 1.1 days and 176.7 ± 102.3 mL, respectively). However, these differences were not significant (OT: p=0.89, FT: p=0.22, LOS: p=1.00, and BL: p=1.00) (Figure 1). For 2-level cases, both groups demonstrated similar OT (NA: 135.7 ± 16.4 vs. RA: 131.2 ± 16.3) and EBL (NA: 210.0 ± 55.2 mL vs. RA: 192.1 ± 94.5 mL). While a lower FT (NA: 70.0 ± 53.0 seconds vs. RA: 45.8 ± 33.4 seconds) and shorter LOS (NA: 1.7 ± 2.3 days vs. RA: 0.9 ± 1.3 days) were also observed in the RA group compared to the NA group, these differences did not reach statistical significance (OT: p=1.00, FT: p=1.00, LOS: p=1.00, and BL: p=0.85) (Figure 2).

Transitional period from NA to RA

**Figure 3:**
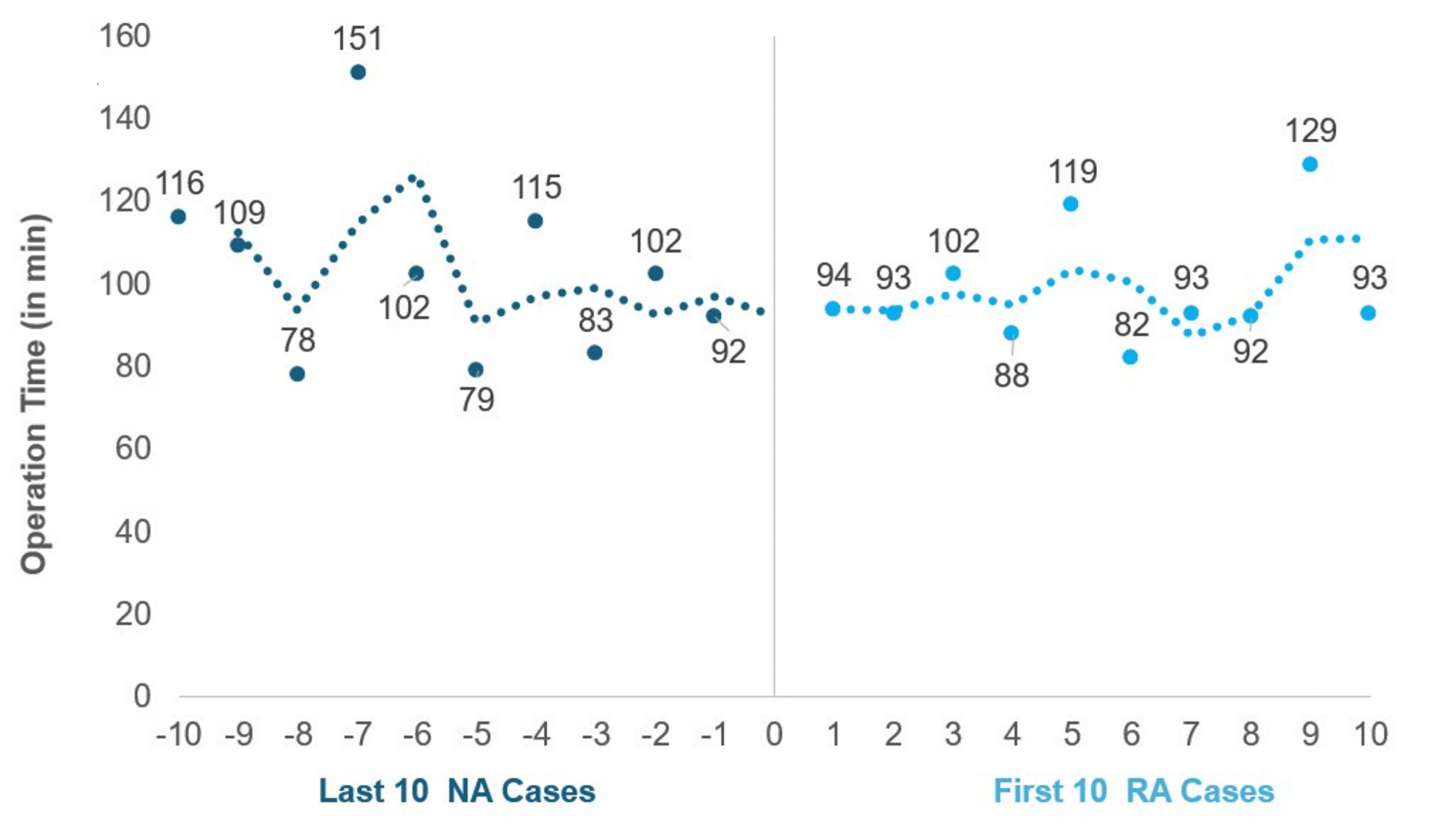
Scatterplots comparing operation time for 1-level open TLIF between the last 10 navigation-assisted (NA) cases and the first 10 robotic-assisted (RA) cases Chronological timeline comparing operation times between the final 10 NA cases (negative x-axis) and the initial 10 RA cases (positive x-axis). TLIF: transforaminal lumbar interbody fusion

**Figure 4:**
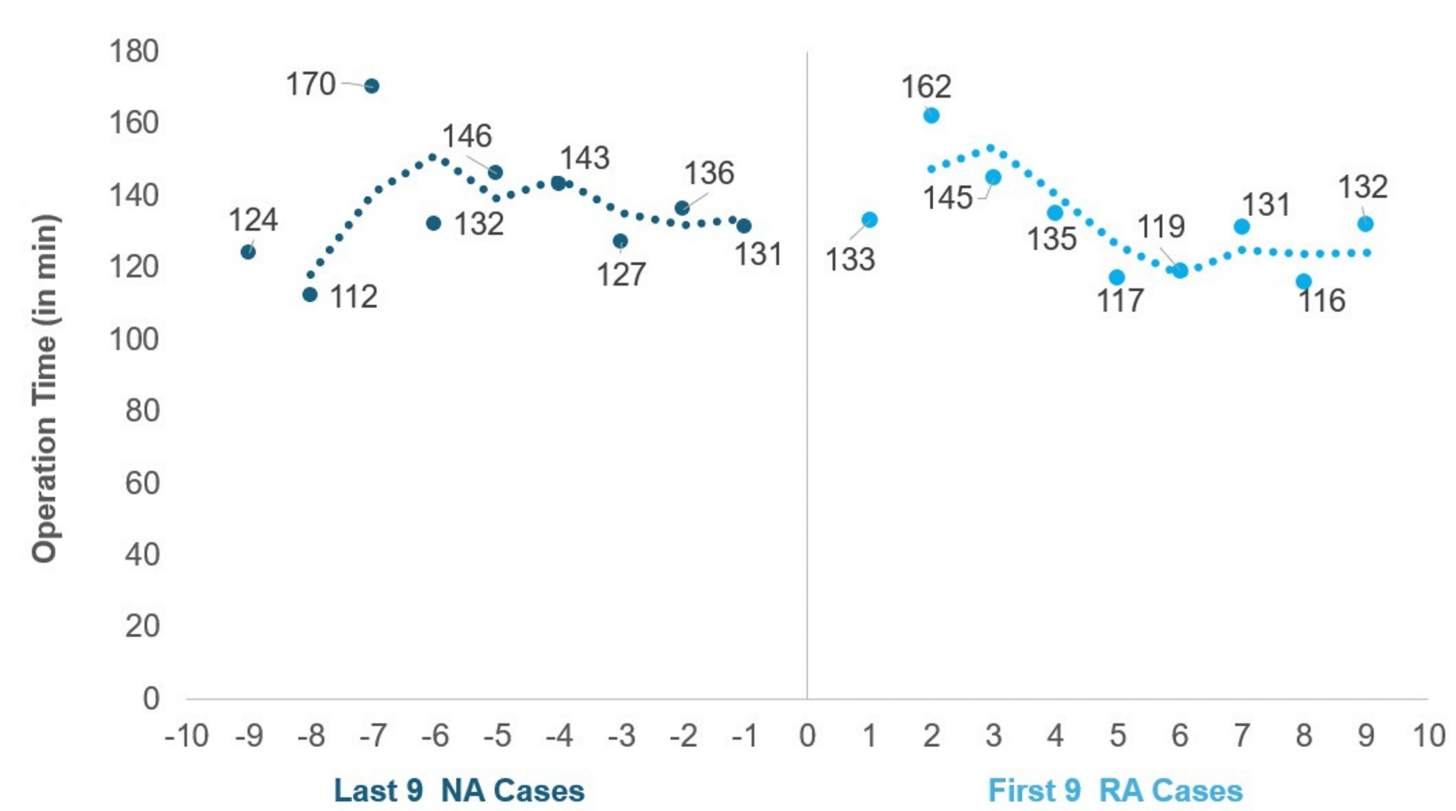
Scatterplots comparing operation time for 2-level open TLIF between the last nine navigation-assisted (NA) cases and the first nine robotic-assisted (RA) cases Chronological timeline comparing operation times between the final nine NA cases (negative x-axis) and the initial nine RA cases (positive x-axis). TLIF: transforaminal lumbar interbody fusion

When looking at the transitional phase from the NA to the RA system, OT, LOS, FT, or EBL were observed between the last 10 NA cases and the first 10 RA cases for 1-level procedures (Table 3). OT was slightly lower in the first 10 RA cases. LOS were very similar. FT trended longer in the last 10 NA cases. EBL trended higher in the first 10 RA cases. Scatterplots of OT of the first 10 RA cases and the last 10 NA cases showed these cases to be similar to each other, with the first 10 RA cases having a narrower range and smaller variance than the last 10 NA cases (Figure 3). For 2-level operations, only nine total consecutive cases were available in the NA group, which were compared to the first nine RA cases (Table 4). OT, FT, and LOS were lower, and EBL was higher in the first nine RA cases than in the last nine NA cases. Scatterplots of OT of the first nine RA cases and the last nine NA cases were similar (Figure 4), though RA cases again had a narrower range and smaller variance.

**Table 3:** Operative efficiency results from the 1-level open TLIF group’s last 10 navigation-assisted (NA) cases and the first 10 robotic-assisted (RA) cases TLIF: transforaminal lumbar interbody fusion; SE: standard error; CI: confidence interval

Parameter	Last 10 NA cases (mean ± SE)	First 10 RA cases (mean ± SE)	First 10 RA - Last 10 NA (95% CI)
Operation time (min)	102.7 ± 7.6	98.5 ± 4.6	-4.2 (-20.3, 11.9)
Fluoroscopy time (sec) (n ≠ 10 as missing data)	70.2 ± 29.8	37.6 ± 7.6 (n=7)	-32.6 (-93.2, 28.0)
Blood loss (mL)	118.5 ± 20.1	210.5 ± 43.8	92.0 (40.0, 144.0)
Length of stay (days)	0.6 ± 0.3	0.7 ± 0.3	0.1 (-0.5, 0.7)

**Table 4:** Operative efficiency results from the 2-level open TLIF group’s last nine navigation-assisted (NA) cases and the first nine robotic-assisted (RA) cases TLIF: transforaminal lumbar interbody fusion; SE: standard error; CI: confidence interval

Parameter	Last 9 NA Cases (mean ± SE)	First 9 RA Cases (mean ± SE)	First 9 RA - Last 9 NA (95% CI)
Operation time (min)	135.7 ± 5.5	132.2 ± 4.9	-3.4 (-8.7, 1.8)
Fluoroscopy time (sec)	70.0 ± 17.7	36.4 ± 6.3	-33.6 (-47.0, -20.1)
Blood loss (mL)	210.0 ± 18.4	228.3 ± 34.1	18.3 (-9.4, 46.1)
Length of stay (days)	1.7 ± 0.8	0.7 ± 0.2	-1.0 (-1.6, -0.4)

Thirty-day revision surgery of the entire study cohort

In the 1- and 2-level open TLIF groups, a total of eight patients required revision surgery. In the 1-level group, four of 170 patients (2.4%) underwent revision, with three of 118 patients (2.5%) in the FA cohort requiring revision for deep wound infections, no revisions in the NA cohort (0 of 20, 0.0%), and one of 32 patients in the RA cohort requiring additional bilateral decompression for a broken osteophyte. In the 2-level group, four of 74 patients (5.4%) underwent revision, all in the FA cohort (4 of 44, 9.1%), including two hematomas and two deep wound infections, while no revisions occurred in the NA cohort (0 of 9, 0.0%) and the RA cohort (0 of 21, 0.0%) required revision. A consolidated summary of 30-day revision surgeries for the study cohort is presented in Table 5.

**Table 5:** Thirty-day revision surgery breakdown after undergoing open TLIF using either fluoroscopy-assisted (FA), navigation-assisted (NA), and robotic-assisted (RA) techniques TLIF: transforaminal lumbar interbody fusion

Procedure	Technique	Count (n) / Total	Occurrence Rate	Reason
1-level	FA	3 / 118	2.5%	Infection (n=3)
	NA	0 / 20	0.0%	-
	RA	1 / 32	3.1%	Additional decompression (n=1)
2-level	FA	4 / 44	9.1%	Hematomas (n=2), infection (n=2)
	NA	0 / 9	0.0%	-
	RA	0 / 21	0.0%	-

## Discussion

There has been a rise in the adoption of advanced technologies to help surgeons place spinal screws more accurately and efficiently. This study evaluates the three main types of guidance systems that are currently used by surgeons: fluoroscopy, navigation, and robotics. Free-hand technique checked with fluoroscopy is the traditional standard, followed by NA, and then RA, which is the latest available system. The goal of this study was to evaluate how adopting NA and RA systems influences operative efficiency as compared to the well-established traditional free-hand technique when performing 1- and 2-level open TLIF procedures. Additionally, this study aimed to evaluate how operative efficiency is impacted when an experienced NA user switches to an RA system during the initial cases.

Operative efficiency was monitored by collecting OT, EBL, LOS, and FT. In general, the RA group showed better operative efficiency when compared to the FA group in both 1- and 2-level TLIF procedures, illustrated by statistically significant (p<0.05) decreases in mean OT, LOS, and EBL. The improved efficiency in the RA group may be attributed to the alignment with the planned screw trajectory provided by the articulating robotic arm, along with the real-time three-dimensional tracking of the instruments while placing the pedicle screws. Additionally, RA may enable less retraction and disruption to surrounding tissues, which may have contributed to lower blood loss. Han et al. examined operative efficiency and found that the RA group had longer OT (by 11 minutes), less blood loss (by 31 mL), more FT (by 10 seconds), and shorter LOS (by 0.13 days) compared to the FA group [18]. Other than OT, all other operative efficiency parameters showed similar trends in this study when compared to Han et al. [18]. The better OT in the RA group observed in this study may be due to the surgeon’s sequential adoption of the FA to the NA to the RA system, rather than transitioning directly from the FA to the RA system, as well as differences in the robotic systems and their usability.

Similar enhancements in operational efficiency were also seen when comparing the NA group and the FA group, where the NA group showed statistically significant (p<0.05) improvements in mean OT and EBL for both 1- and 2-level procedures. Additionally, reductions in LOS were noted with NA compared to FA, which were found to be significantly different (p<0.05) in 1-level procedures. When evaluating OT in 105 patients undergoing L5-S1 spinal fusion for treatment of isthmic spondylolisthesis with pedicle screws placed using either an NA or FA system, Sasso et al. showed the NA system yielded lower OT than the FA system [19,20]. It is believed that the three-dimensional tracking of the instruments with respect to the registered image enables surgeons to maintain clear visualization during screw placement, generally allowing a more accurate and efficient surgery without needing to pause for real-time images. Lastly, this study showed that there was no statistical difference in operative efficiency between the RA and NA systems for both 1- and 2-level procedures. Al-Naseem et al. found a similar pattern in their study, where there was no significant difference in perioperative outcomes between RA and NA groups, which may be because both systems feature similar preoperative planning and real-time three-dimensional instrument navigation capabilities that allow surgeons to plan and place spinal screws accurately [21].

Many of the surgeons started their careers with the FA system and then switched to either the NA or RA systems. Previous studies have shown the learning curve for an FA user to switch to a NA system to be around 25 cases and 20-30 cases to switch to a RA system [22,23]. This initial period of learning required to switch to a new system may increase surgeons' hesitation to adopt the RA system when they are currently using an NA system, due to perceived loss of efficiency. Furthermore, NA and RA are generally characterized together as computer-aided surgery, which makes it challenging in the current literature to distinguish between the two when looking at data and the learning curve. This study observed no immediate impact on operational efficiency when a user transitioned from an NA to RA system, as evident by similar OT and LOS results between the first 10 RA cases and the last 10 NA cases in the 1-level group. Therefore, we have demonstrated that surgeons can still perform efficient surgery in an outpatient setting, including 1- and 2-level open TLIFs, even with RA, with LOS averaging less than one day. However, further studies with a larger number of patients and more contributing surgeons may provide a better understanding of the adoption among different types of assisted systems.

The 30-day postoperative revision data showed that the FA group had the highest rate of revision surgeries compared to the NA and RA groups for both 1- and 2-level open TLIF procedures. This trend aligns with findings from previous studies demonstrating lower complication rates with the use of NA and RA systems [24,25]. These improved outcomes may be attributed to reduced soft tissue disruption and the enhanced safety and accuracy afforded by real-time instrument tracking and rigid arm guidance, which facilitate more accurate screw placement and minimize intraoperative error.

There are some noteworthy limitations of this study. First, it is retrospective in design, introducing potential selection bias and limiting causal interpretation. Second, all procedures were performed by a single surgeon at a single site, which may reduce the generalizability of the findings. Third, even though the intent of the study was to evaluate surgical efficiency and adoption barriers of enabling technologies, the short follow-up period of four weeks postoperatively limits the assessment of long-term outcomes. These include fusion rates and delayed complications, which are important indicators of procedural durability and overall patient recovery. Additionally, this study analyzed 10 years of operative data; thus, the efficiency of the NA and RA surgeries, which occurred late in the chronological series, is likely benefiting from general innovations in improved anesthesia recovery, better nursing familiarity with the surgeon's workflow, and more advanced perioperative enhanced recovery after surgery (ERAS) protocols developed over time and experience. Furthermore, this study received external financial support from Globus Medical, Inc.; thus, the potential for bias inherent in industry-supported research is acknowledged. To minimize such bias, all data analysis was conducted independently, and findings are reported without external input.

## Conclusions

Surgical robotics and navigation systems are being more widely adopted for the placement of spinal screws because they have been observed to increase screw placement accuracy and reduce screw-related complications compared to the well-established free-hand fluoroscopy techniques. Preoperative planning, along with intraoperative guidance for screw trajectories, are aspects of these systems that may allow surgeons to confidently execute a surgical plan with high accuracy and efficiency. This study analyzed the operative efficiency among the FA, NA, and RA cohorts of patients who underwent 1- and 2-level open TLIFs by a single surgeon. There was no significant difference between the NA and RA systems, but each demonstrated statistically significant reductions compared to the FA system in terms of OT, LOS, and EBL, with similar observations in 1- and 2-level surgeries. An additional notable finding was that there was no change in OT and LOS in the transitional period when an experienced NA system user switched to an RA system.
